# Beyond Drinking-Age Thresholds: Alcohol Misuse and Alcohol-Related Harms Among University Students in Trinidad and Tobago

**DOI:** 10.7759/cureus.114408

**Published:** 2026-08-12

**Authors:** Adrian H Palmer, Vasant Basdeo, Shastri Motilal, Raveed Khan, Rohan G Maharaj

**Affiliations:** 1 Sport and Recreation, University of Trinidad and Tobago, Arima, TTO; 2 Family Medicine, Holistic Medical Centre, Penal, TTO; 3 Paraclinical Sciences, The University of the West Indies, St. Augustine, TTO; 4 Family Medicine, The University of the West Indies, St. Augustine, TTO; 5 Technical Advisor, Healthy Caribbean Coalition, Christ Church, BRB

**Keywords:** alcohol drinking, alcohol misuse, binge drinking, students, trinidad and tobago, universities

## Abstract

Objective

This study aimed to examine the prevalence and patterns of alcohol use and misuse among undergraduate students aged 18-25 years in Trinidad and Tobago and to assess whether students younger than 21 years were more likely to report alcohol misuse and alcohol-related harms.

Methods

A quantitative cross-sectional design was used, involving a secondary analysis of two harmonized surveys conducted at the University of Trinidad and Tobago (UTT) and The University of the West Indies, St. Augustine (UWI). The analytic sample included 800 undergraduate students aged 18-25 years, who were selected through multistage sampling. Alcohol misuse was defined as binge drinking or heavy drinking during the past 30 days. Analyses included descriptive statistics, chi-square tests of homogeneity to assess differences in prevalence, and binary logistic regression to identify factors associated with current alcohol misuse.

Results

Current alcohol use was reported by 60.8% of students with valid responses (471/775; 95% confidence interval (CI): 57.3-64.2), and 29.1% (225/772; 95% CI: 26.0-32.4) of students with valid responses met the criteria for alcohol misuse. Students aged 21-25 years had significantly higher odds of alcohol misuse than those aged 18-20 years (adjusted odds ratio (aOR): 1.50, standard error (SE) = 0.19, 95% CI: 1.03-2.17, p = 0.034). Male students had similar odds of alcohol misuse compared with female students (aOR: 1.07, SE = 0.189, 95% CI: 0.74-1.55, p = 0.725). Lifetime alcohol use was reported by 93.0% (744/800; 95% CI: 91.1-94.6) of students. Among UWI students, 82.5% (254/308) of students with valid responses reported initiating alcohol use during primary or secondary school. Among UTT students, selected alcohol-related harms, including unprotected sex (p = 0.032) and driving under the influence (p < 0.001), were more prevalent among students aged 21-25 years than among those aged 18-20 years.

Conclusions

Undergraduate students aged 21-25 years were at greater risk of alcohol misuse and selected alcohol-related harms, including driving under the influence and unprotected sex, than students aged 18-20 years. Female students had similar odds of alcohol misuse compared with male students. The findings also suggest a high prevalence of early alcohol initiation among undergraduates in Trinidad and Tobago. These results support the need for public health interventions that extend beyond legal drinking-age thresholds.

## Introduction

Alcohol consumption is deeply embedded in the cultural fabric of Trinidad and Tobago, serving as a “social lubricant” widely accepted among the youth [1]. The Trinidad and Tobago Non-Communicable Diseases Risk Factor Survey 2024 indicated that the 18-29-year-old cohort exhibited the second-highest rates of current alcohol use and heavy episodic drinking [2]. The Government of Trinidad and Tobago has indicated its intention to raise the legal drinking age from 18 to 21 to address underage drinking, as well as public health and safety concerns [3]. Alcohol has no safe level of consumption; it is a psychoactive, toxic, and carcinogenic substance with dependence-inducing properties and contributes to the burden of over 200 diseases and injuries [4,5]. The Sustainable Development Goal (SDG) target 3.5 emphasizes the prevention of harmful alcohol consumption and associated negative consequences [5].

The National Alcohol Survey of Households in Trinidad and Tobago (NASHTT) revealed that households with alcohol consumers and heavy episodic drinkers were significantly more likely to report relationship and behavioural issues, lifestyle-related diseases, traffic offenses, and the promotion of youth alcohol consumption [6]. Approximately 80% of households support raising the legal drinking age to 21 and holding alcohol sellers accountable for sales to underage individuals [7]. University students in Trinidad and Tobago have reported alcohol-related negative effects, including missing classes, cravings, and interpersonal issues [8]. Students in other jurisdictions have reported similar effects, including poor academic performance, general and sexual health issues, interpersonal conflicts, and driving while under the influence [9-11].

The United States is among the few countries with a legal drinking age of 21. This policy has been associated with a lower likelihood of substance use disorder, a 20-30% reduction in alcohol use, and a 10% reduction in fatal road traffic accidents among male adults [12,13]. However, increased alcohol access at age 21 has also been linked to higher rates of medical care seeking for alcohol overdose and associated injuries [14]. Therefore, policies raising the legal drinking age should be supported by complementary interventions to address potential unintended consequences, including persistent alcohol misuse and underage access through alternative sources [15].

Understanding alcohol-use patterns among young adults in Trinidad and Tobago, particularly differences between students aged 18-20 years and those aged 21-25 years, may provide useful baseline evidence for public health planning and future alcohol policy evaluation. This study assessed the association between age and alcohol misuse and selected alcohol-related harms among undergraduate students aged 18-25 years.

This work was previously presented as an oral presentation at the 10th Caribbean Alcohol Reduction Day (CARD 2026) on January 29, 2026.

## Materials and methods

Study design

This study involved a secondary analysis of harmonized data derived from two independently conducted, anonymous cross-sectional surveys. Both surveys required participants to provide informed consent before participation. The present study was conducted as a collaborative analysis involving authors affiliated with the University of Trinidad and Tobago (UTT), the University of the West Indies, St. Augustine (UWI), the Holistic Medical Centre, and the Healthy Caribbean Coalition.

The UTT dataset was derived from a study conducted by authors affiliated with UTT and the Healthy Caribbean Coalition. The UWI dataset was derived from a separate study conducted collaboratively by authors affiliated with the Holistic Medical Centre and UWI. The present study involved harmonizing the two datasets and jointly analyzing alcohol use, alcohol misuse, and alcohol-related harms among undergraduate students aged 18-25 years in Trinidad and Tobago.

Data sources

Two independently collected datasets were used. The UWI dataset was collected between October and November 2023 from undergraduate students aged 18-25 years at UWI and examined vaping and other substance use behaviours, including alcohol consumption. The UTT dataset was collected in February 2025 from undergraduate students aged 18-25 years at UTT and focused on alcohol consumption patterns and substance use practices.

Sampling strategy

The combined sample comprised 800 undergraduate students from UTT (465) and UWI (335). UTT required a minimum sample size of 359, which was determined using a 95% confidence level, 5% margin of error, and a 50% population proportion, based on an estimated undergraduate population of 5,346 for Semester 1, 2024-2025. UWI required a minimum sample size of 219, which was calculated using a 95% confidence level, 5% margin of error, and a 17.2% population proportion, referencing the 2017 Global Youth Tobacco Survey prevalence of e-cigarette use among students aged 13-15 years [16]. The undergraduate population at UWI was estimated to be 11,299 based on the latest available enrollment data, which were from the 2019-2020 academic year.

Data from undergraduate students aged 18-25 years enrolled at UTT and UWI were eligible for inclusion in the study. Data collected from UTT excluded students if they reported abstaining from alcohol consumption in the past 30 days due to medical advice or if they were blind and/or deaf. No additional exclusion criteria were applied to the UWI dataset beyond the study eligibility requirements.

Sampling method

A multistage sampling approach was used for both institutional datasets. The UTT survey initially involved quota sampling to ensure proportional representation across the nine campuses. During campus visits, available classes were then selected using convenience sampling because class schedules were unavailable for random sampling. Eligible students within each selected class were assigned numbers, and a randomizer was used to select 60% of students for participation. The final UTT sample was reviewed for proportionality across campuses.

**Figure 1:**
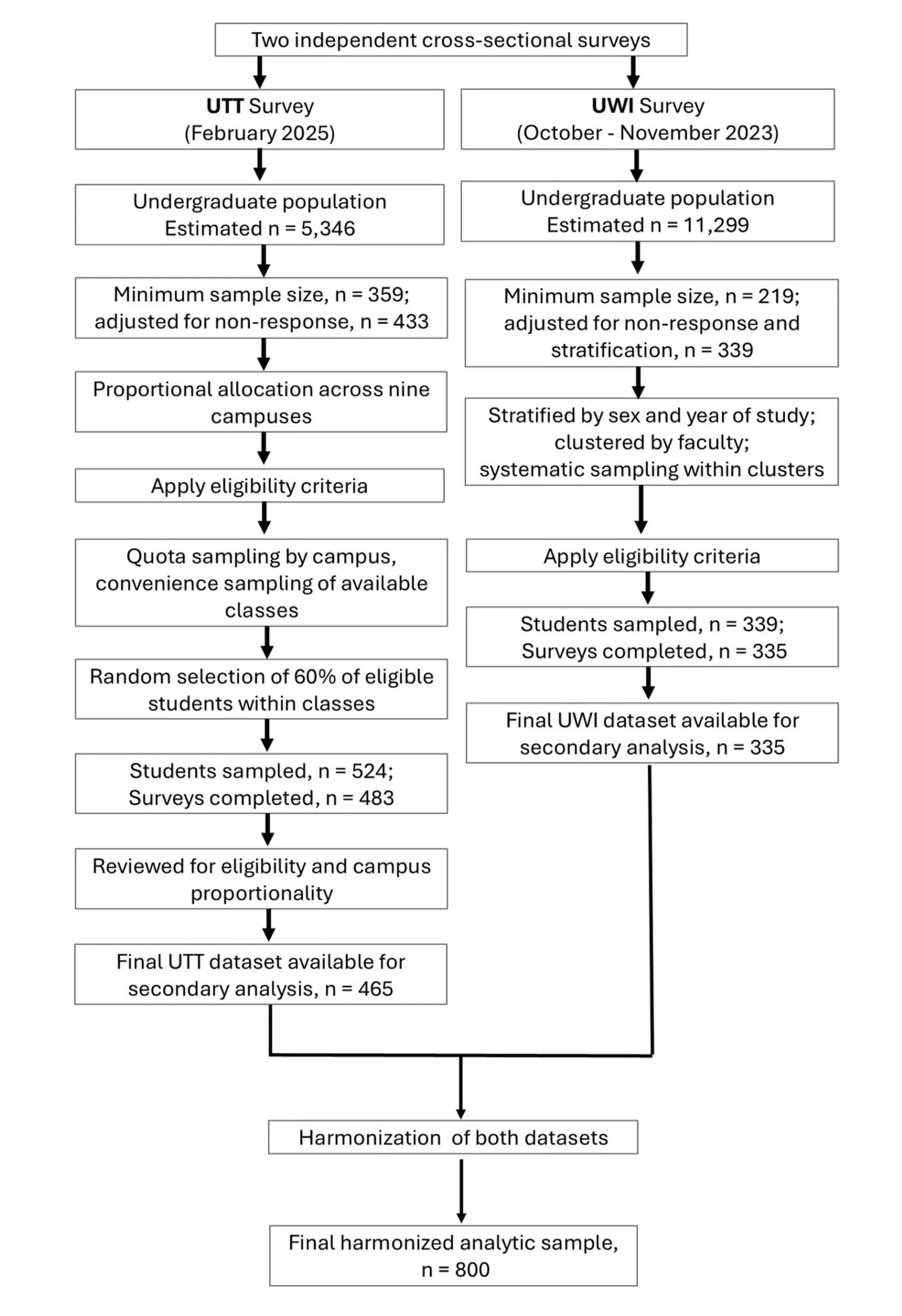
Flowchart depicting student selection for the final harmonized analytic sample UTT: The University of Trinidad and Tobago; UWI: The University of the West Indies, St. Augustine

The UWI survey employed a structured probabilistic sampling design to support representative coverage across eight faculties. This approach incorporated proportional stratified sampling by sex and year of study, cluster sampling by faculty, and systematic sampling within the selected clusters. The student selection process for both institutional datasets, including sample size calculations, sampling procedures, numbers of completed surveys, and the final datasets available for secondary analysis, is summarized in Figure 1.

Data integration and management

The UTT and UWI surveys were independently designed, and variables were harmonized where they were directly or conceptually comparable. The original measures, harmonized coding rules, non-equivalent elements, and rationale for comparability are summarized in Table 1. Both studies defined a standard drink as containing 14 grams (0.6 ounces) of pure alcohol, equivalent to 12 oz. of beer (5%), 8 oz. of malt liquor (12%), 5 oz. of wine (12%), or 1.5 oz. of distilled spirits (40%) [17]. Both datasets captured alcohol consumption patterns over the past 30 days. Participants who reported no drinking during this period were classified as abstainers. Moderate drinking was defined slightly differently in each dataset. The UTT consumption patterns were guided by the National Institute on Alcohol Abuse and Alcoholism [18]; the UWI dataset used a weekly consumption threshold for moderate drinking. Therefore, moderate drinking was considered conceptually aligned but not fully equivalent across datasets and was interpreted descriptively. The primary harmonized outcome, alcohol misuse, was derived from binge drinking and/or heavy drinking in the past 30 days rather than from moderate drinking.

**Table 1:** Variable harmonization framework for UTT and UWI datasets UTT: The University of Trinidad and Tobago; UWI: The University of the West Indies, St. Augustine Campus

Variable	UTT measure	UWI measure	Harmonized dataset measure	Missing and non-equivalent data	Harmonized coding rule	Rationale
Age	Numeric age, 18-25 years	Numeric age, 18-25 years	Numeric age, 18-25 years	Directly comparable	Same variable and values	-
Age group			Two values: 18-20 years, 21-25 years	Not applicable	Recoded from the age variable and categorized as 18-20 and 21-25 years	These age categories were selected to compare alcohol-use patterns between students younger than 21 years and those aged 21 years or older
Sex	Two values: male, female	Two values: male, female	Two values: male, female	Directly comparable	Same variable and values	-
Ethnicity	Five values: Afro-Caribbean, Indo-Caribbean, mixed, Caucasian, other	Five values: Afro-Caribbean, Indo-Caribbean, mixed, Caucasian, other	Four values: Afro-Caribbean, Indo-Caribbean, mixed, other	Caucasian missing	Caucasian was recoded as "other"	There was a total of two respondents who indicated Caucasian. This low number affected statistical analysis; hence, it was incorporated into "other"
Religious affiliation	Six values: Christian, Seventh Day Adventist, Hinduism, Islam, other, none	Five values: Christian, Hinduism, Islam, other, none	Five values: Christian, Hinduism, Islam, other, none	Seventh Day Adventist missing	Seventh Day Adventist from UTT was recoded to Christian	Seventh Day Adventist is a Christian denomination; however, UWI did not have a Seventh Day Adventist
Relationship status	Six values: single, romantic relationship (boyfriend or girlfriend), common-law/cohabitational relationship, married, divorced/separated, widowed/widower	Four values: married/common-law, single, widowed/widower, divorced/separated	Two values: single, committed	Single in the UWI dataset included persons who would have been considered to be in a romantic relationship (boyfriend or girlfriend) in the UTT dataset	Married and cohabiting/common-law relationship values were recoded to “committed”. Romantic relationship, divorced/separated, widow/widower were recoded to “single”. In both UWI and UTT datasets, “single” referred to persons who were not married and not cohabiting	To harmonize the data, romantic relationship (boyfriend or girlfriend) in the UTT dataset was recoded to "single." Married and common-law/cohabitational relationships were recoded together as “committed” since these values had a low frequency, totalling 14. Widow/widower had a frequency of zero across datasets, and Divorced/Separated had a total frequency of two and were recoded as "single"
Lifetime alcohol use	Have you ever consumed a drink of alcohol (or drunk any alcoholic beverage), even just once? Values: yes, no	Have you ever drunk alcohol, even just once? values: yes, no	Lifetime alcohol use; values: yes, no	Directly comparable	Same variable and values	-
Current alcohol use	During the past 30 days, did you consume any alcoholic beverages, even once? Two values: yes, no	During the last 30 days, how many days, if any, did you drink alcohol? Seven values: every day, 20-29 days, 10-19 days, 6-9 days, 3-5 days, 1 or 2 days, none	Current alcohol use (past 30 days): two values: yes, no	Conceptually are comparable	For the UWI dataset, the following values were recoded to "yes": every day, 20-29 days, 10-19 days, 6-9 days, 3-5 days, 1 or 2 days. None was recoded to "no"	This was done to make the UWI measurement similar to that of UTT: “during the past 30 days, did you consume any alcoholic beverages, even once”
Abstainer	Which of the following options BEST describes your consumption of alcoholic beverages within the past 30 days: value: did not consume any alcohol	The option which best describes the amount of alcohol you consumed in the last 30 days, if any at all: value: I did not drink alcohol	Abstainer (past 30 days); values: yes, no	Directly comparable	Those who indicated the respective value in each dataset were coded “yes”, and those who indicated other values were coded “no”	-
Moderate drinking	Which of the following options BEST describes your consumption of alcoholic beverages within the past 30 days: value: men: 2 or less drinks in a day; women: 1 or less in a day	The option which best describes the amount of alcohol you consumed in the last 30 days, if any at all. Value: females: less than 7 drinks per week	Moderate drinking (past 30 days); values: yes, no	Not fully equivalent, but comparable to an extent	Those who indicated the respective value in each dataset were coded “yes”, and those who indicated other values were coded “no”	In the UWI data set, a person drinking less than 7 drinks per week was considered moderate drinking. In the UTT data, persons who had this consumption would have met the criteria for moderate drinking
Heavy drinking	Which of the following options BEST describes your consumption of alcoholic beverages within the past 30 days: value: men: 5 or more drinks on a day or 15 or more drinks per week; women: 4 or more drinks on a day or 8 or more drinks per week	The option which best describes the amount of alcohol you consumed in the last 30 days, if any at all. Value: “(female) 8 or more drinks per week; male) 15 or more drinks per week”	Heavy drinking (past 30 days): values: yes, no	Directly comparable	Those who indicated the respective value in each dataset were coded “yes”, and those who indicated other values were coded “no”	-
Binge drinking	Which of the following options BEST describes your consumption of alcoholic beverages within the past 30 days: value: men: 5 or more drinks consumed in a single occasion (within 2 hours); women: 4 or more drinks consumed in a single occasion (within 2 hours)	In the last 30 days, did you ever consume 5 drinks or more in a couple of hours? Values: yes, no	Binge drinking (past 30 days): values: yes, no	Conceptually comparable	Those who indicated the value in the UTT dataset were coded “yes”, and those who indicated other values were coded “no”. Those who were coded as “yes” or “no” in the UWI dataset remained “yes” or “no” in the harmonized dataset	A female student who consumed 4 drinks within a couple of hours at UTT would have been determined a binge drinker. However, in the UWI dataset, it needed to be 5 or more. Though a subjective difference of 1 drink, both were designed to describe a binge drinker
Alcohol misuse	Variable not used in the UTT study	Variable not used in the UWI study	Binge drinking (past 30 days): values: yes, no		In the UWI dataset, based on how questions were asked, a person could indicate both moderate drinking and heavy or binge drinking. However, once a person also indicated binge or heavy drinking, their overall past 30-day pattern was described as alcohol misuse	Heavy and binge drinking were categorized as alcohol misuse (in keeping with the National Institute on Alcoholism and Alcohol Abuse recommendations). Alcohol misuse was not an original variable in either dataset; it was derived for the present analysis from binge drinking and/or heavy drinking responses

Certain variables were analyzed separately by dataset due to differences in data collection. The UTT dataset included scores from the World Health Organization-approved AUDIT, ranging from 0 to 40, with thresholds indicating abstention, low-risk use, hazardous use, or likely alcohol dependence [19]. UTT also included self-reported negative effects of alcohol use experienced within the past 12 months. The UWI dataset, in contrast, incorporated additional variables such as age and the educational level at which alcohol use was first initiated.

Ethical considerations

The UTT study obtained ethical approval from both the UWI, St. Augustine Campus Research Ethics Committee (CREC-SA.2880/10/2024) and the UTT Research Ethics Committee (UTT-T/08/25). The UWI study was approved by the UWI, St. Augustine Campus Research Ethics Committee (CREC-SA.2289/08/2023). Data collection was conducted by the authors of the present study; all datasets contained anonymized participant information, ensuring confidentiality. Furthermore, data-sharing agreements were established among the researchers involved in the study.

Statistical analysis

All statistical analyses were performed using IBM SPSS Statistics version 30.0 (IBM Corp., Armonk, NY), with a significance threshold set at p ≤ 0.05. Analyses were conducted as unweighted analyses of the harmonized analytic sample. Binary logistic regression models were applied to identify sociodemographic predictors of alcohol misuse. Independent variables included age group, institution, current alcohol use, sex, ethnicity, religion, and relationship status. The dependent variable was dichotomous, and all predictors were categorical; therefore, assessment of linearity in the logit for continuous predictors was not applicable. Observations were independent, with no paired or repeated measurements included. Multicollinearity was assessed using tolerance and variance inflation factor (VIF) values. Tolerance values ranged from 0.914 to 0.987, and VIF values ranged from 1.013 to 1.094, indicating no evidence of problematic multicollinearity. Model performance was summarized using Nagelkerke R² and classification accuracy. Adjusted odds ratios (aORs) along with their corresponding 95% confidence intervals (CIs) were reported to quantify associations.

Although the parent surveys used multistage sampling approaches, the combined dataset did not include sufficient design information to construct consistent survey weights or apply design-based variance estimation across both institutions. Therefore, CIs, chi-square tests, and logistic regression results should be interpreted as unweighted sample estimates rather than design-weighted population estimates. Missing data were handled using available-case analysis for descriptive estimates. Participants were included in each analysis if they had valid responses for the variables required for that specific analysis. Therefore, denominators varied across variables and are reported where applicable. Percentages were calculated using valid responses for each variable. However, for the binary logistic regression, model-specific listwise deletion was applied.

## Results

Data from 800 undergraduate students aged 18 to 25 years were included in this study, with participants recruited from Trinidad and Tobago’s two largest universities. Females represented a higher proportion of the sample, accounting for 53.8% (430/799) of valid responses. Among students with valid responses, current alcohol consumption within the past 30 days was reported by 60.8% (471/775; 95% CI: 57.3-64.2) of students, while 29.1% (225/772; 95% CI: 26.0-32.4) of students met the criteria for alcohol misuse. Data from UTT, assessed using the AUDIT, indicated that 3.3% (15/457; 95% CI: 1.9-5.2) of students with valid responses were classified as likely alcohol dependent or as having moderate to severe alcohol use disorder (AUD); of these students, 60.0% (9/15) were older than 20 years. The confidence intervals presented in Table 2 indicate that the sample of UWI students demonstrated a higher prevalence of current alcohol use and misuse, alongside a lower prevalence of abstinence, compared to UTT.

**Table 2:** Sociodemographic characteristics and alcohol consumption patterns of undergraduate students aged 18-25 in Trinidad and Tobago, 2023-2025 Percentages are calculated using valid responses for each variable. Missing data varied by variable. Valid responses were available for current alcohol use in 775 of 800 students, alcohol misuse in 772 of 800 students, and lifetime alcohol use in 800 of 800 students UTT: The University of Trinidad and Tobago; UWI: The University of the West Indies, St. Augustine; CI: confidence interval

	Overall		UTT		UWI	
Sociodemographic factors	N (%)	95% CI	N (%)	95% CI	N (%)	95% CI
Students	800 (100)	-	465 (58.1)	-	335 (41.9)	-
Age group, years	-	-	-	-	-	-
18-20	420 (52.5)	49.0-55.9	270 (58.1)	53.5-62.5	150 (44.8)	39.5-50.1
21-25	380 (47.5)	44.1-51.0	195 (41.9)	37.5-46.5	185 (55.2)	49.9-60.5
Sex	-	-	-	-	-	-
Male	369 (46.2)	42.7-49.6	241 (51.9)	47.4-56.5	128 (38.2)	33.1-43.5
Female	430 (53.8)	50.4-57.3	223 (48.1)	43.5-56.5	207 (61.8)	56.5-66.9
Ethnicity	-	-	-	-	-	-
Afro-Caribbean	266 (33.3)	30.1-36.6	168 (36.2)	31.9-40.7	98 (29.3)	24.6-34.3
Indo-Caribbean	277 (34.7)	31.4-38.0	136 (29.3)	25.3-33.6	141 (42.1)	36.9-47.4
Mixed	238 (29.8)	26.7-33.0	144 (31.0)	27.0-35.4	94 (28.1)	23.5-33.0
Other	18 (2.3)	1.4-3.5	16 (3.4)	2.1-5.4	2 (0.6)	0.1-1.9
Religious affiliation	-	-	-	-	-	-
Christianity	510 (64.2)	60.8-67.4	303 (65.3)	60.9-69.5	207 (62.5)	57.2-67.6
Hinduism	129 (16.2)	13.8-18.9	56 (12.1)	9.3-15.3	73 (22.1)	17.8-26.8
Islam	49 (6.2)	4.6-8.0	34 (7.3)	5.2-10.0	15 (4.5)	2.7-7.2
Other	42 (5.3)	3.9-7.0	41 (8.8)	6.5-11.7	1 (0.3)	0.0-1.4
No religious affiliation	65 (8.2)	6.4-10.2	30 (6.5)	4.5-9.0	35 (10.6)	7.6-14.2
Relationship status	-	-	-	-	-	-
Single	783 (98.2)	97.1-99.0	451 (97.4)	95.7-98.6	332 (99.4)	98.1-99.9
Committed	14 (1.8)	1.0-2.9	12 (2.6)	1.4-4.3	2 (0.6)	0.1-1.9
Lifetime alcohol use	744 (93.0)	91.1-94.6	432 (92.9)	90.3-95.0	312 (93.1)	90.1-95.5
Alcohol consumption pattern (30-day)	-	-	-	-	-	-
Current alcohol use	471 (60.8)	57.3-64.2	256 (55.2)	50.6-59.7	215 (69.1)	63.8-74.1
Abstainer	299 (38.7)	35.3-42.2	200 (43.5)	39.0-48.0	99 (31.7)	26.8-37.0
Moderate drinking	248 (32.1)	28.9-35.5	147 (32)	27.8-36.3	101 (32.4)	27.4-37.7
Alcohol misuse	225 (29.1)	26.0-32.4	113 (24.6)	20.8-28.7	112 (35.9)	30.7-41.3

Lifetime alcohol use was reported by 93.0% of students (744/800; 95% CI: 91.1-94.6). Among UWI students with valid responses, 82.5% (254/308) reported initiating alcohol use during primary or secondary school. Most UWI students initiated alcohol use during secondary school, with 66.6% (205/308) reporting initiation during this period, corresponding to ages 11-18 years. Initiation during primary school was reported by 15.9% (49/308) of students, while 17.5% (54/308) of students reported first using alcohol during tertiary education.

Comparative analysis of students aged 18-20 and 21-25 years in the sample, as presented in Table 3, indicated that the prevalence of alcohol misuse was significantly higher among students aged 21-25 years, at 34.4% (126/366; 95% CI: 29.7-39.4), compared to 24.4% (99/406; 95% CI: 20.4-28.7) among those aged 18-20 years. Among current drinkers, alcohol misuse was reported by 95 of 230 students aged 18-20 years (41.3%; 95% CI: 35.1-47.7) and 123 of 239 students aged 21-25 years (51.5%; 95% CI: 45.1-57.8). This difference was statistically significant, χ²(1, N = 469) = 4.86, p = 0.027, corresponding to an odds ratio of 1.51 (95% CI: 1.05-2.17).

**Table 3:** Age-based comparison of alcohol consumption patterns of undergraduate students aged 18-25 in Trinidad and Tobago, 2023-2025 ^*^Chi‑square test of homogeneity used to assess differences in percentages across age groups. ^**^Consumption patterns that showed significantly different percentages across age groups Percentages are calculated using valid responses for each variable; some variables had missing data CI: confidence interval

	18-20 years		21-25 years			
Consumption pattern (30-day)	N (%)	95% CI	N (%)	95% CI	χ2(df)	P-value^*^
Students	406	-	380	-	-	-
Current alcohol use	230 (56.4)	51.5-61.1	241 (65.7)	60.7-70.4	7.002(1)	0.008^**^
Abstainer	172 (42.4)	37.6-47.2	127 (34.7)	30.0-39.7	4.835(1)	0.028^**^
Moderate drinking	135 (33.3)	28.8-37.9	113 (30.9)	26.3-35.7	0.540(1)	0.462
Alcohol misuse	99 (24.4)	20.4-28.7	126 (34.4)	29.7-39.4	9.070(1)	0.003^**^

Binary logistic regression was performed, adjusting for current alcohol use, age group, institution, sex, relationship status, ethnicity, and religious affiliation. Age group was significantly associated with current alcohol misuse. As shown in Table 4, students aged 21-25 years were 50% more likely to misuse alcohol than those aged 18-20 years, with an adjusted odds ratio of 1.50 (95% CI: 1.03-2.17). The UTT and UWI datasets were separately analyzed using the same regression model, and the association between age group and current alcohol misuse was consistent across the pooled and institution-specific analyses. The UTT-specific model yielded an aOR of 1.47 (95% CI: 0.89-2.45), while the UWI-specific model yielded an aOR of 1.50 (95% CI: 0.86-2.63). Although the age-group association was not statistically significant in the institution-specific analyses, the similarity of the effect estimates suggests that the observed association was not driven by the harmonization of the two datasets.

**Table 4:** Sociodemographic factors and their association with current (30-day) misuse of alcohol among undergraduate students aged 18-25 in Trinidad and Tobago, 2023-2025 ^*^P-values produced from a binary logistic regression model. ^**^Statistically significant p-values The binary logistic regression model explained 37.2% of the variance in alcohol misuse (Nagelkerke R²) and correctly classified 75.2% of cases overall. The model identified students who misused alcohol with 46.6% accuracy and correctly classified non‑misusers with 87.0% accuracy. Alcohol misuse was observed in 225 students with valid responses. The final logistic regression model included 12 predictor parameters, yielding approximately 18.8 outcome events per parameter. This was considered sufficient for the planned regression analysis based on commonly used events-per-parameter guidance. The final binary logistic regression model included 763 of 800 participants (95.4%); 37 participants (4.6%) were excluded because of missing data on alcohol misuse or one or more predictor variables The first value under each variable is the reference value OR: odds ratio; SE: standard error; CI: confidence interval; UTT: The University of Trinidad and Tobago; UWI: The University of the West Indies, St. Augustine

Variable	Adj. OR	SE	95% CI	P-value^*^
Age group, years	-	-	-	-
18-20	-	-	-	-
21-25	1.5	0.19	1.03-2.17	0.034^**^
Sex	-	-	-	-
Females	-	-	-	-
Males	1.07	0.189	0.74-1.55	0.725
Relationship status	-	-	-	-
Committed	-	-	-	-
Single	0.9	0.725	0.22-3.72	0.883
Institution	-	-	-	-
UTT	-	-	-	-
UWI	1.2	0.197	0.82-1.77	0.346
Current drinker	-	-	-	-
No	-	-	-	-
Yes	33.94	0.396	15.61-73.79	<0.001^**^
Ethnicity	-	-	-	-
Afro-Caribbean	-	-	-	-
Indo-Caribbean	1.22	0.271	0.72-2.07	0.472
Mixed	0.82	0.236	0.52-1.31	0.405
Other	0.69	0.66	0.19-2.52	0.574
Religious affiliation	-	-	-	-
None	-	-	-	-
Christianity	0.6	0.341	0.31-1.16	0.129
Hinduism	0.89	0.405	0.40-1.98	0.783
Islam	0.35	0.573	0.11-1.07	0.348
Other	0.8	0.499	0.30-2.14	0.662

As presented in Table 5, UTT students aged 21-25 years exhibited higher prevalence rates for all alcohol-related negative effects experienced in the past 12 months, except for injury; however, most of these differences were not statistically significant. Notably, unprotected sex and driving under the influence were significantly associated with age group, with higher rates observed among students aged 21-25 years.

**Table 5:** Age-based comparison of the negative effects alcohol consumption experienced by undergraduate students aged 18-25 in UTT, 2025 ^*^Chi‑square test of homogeneity used to assess differences in percentages across age groups. ^**^Indicates alcohol‑related negative effects that showed significantly different prevalence rates across age groups The UTT sample included 465 students, with 270 aged 18–20 years and 195 aged 21–25 years. Percentages are calculated using valid responses for each variable; some variables had missing data UTT: The University of Trinidad and Tobago; CI: confidence interval

	18-20 years		21-25 years		-	-
Alcohol negative effects (past 12 months)	N (%)	95% CI	N (%)	95% CI	χ2(df)	P-value^*^
General health	-	-	-	-	-	-
Became injured	18 (6.7)	4.2-10.2	8 (4.1)	2.0-7.6	1.424(1)	0.233
Emergency medical care	2 (0.7)	0.2-2.4	5 (2.6)	1.0-5.6	2.563(1)	0.135
Hangover or feeling ill	99 (36.7)	31.1-42.5	71 (37)	30.4-44.0	0.005(1)	0.945
Sexual health	-	-	-	-	-	-
Sexual coercion	6 (2.2)	0.9-4.5	8 (4.1)	2.0-7.6	1.378(1)	0.24
Sexually transmitted infections	1 (0.4)	0.0-1.7	3 (1.5)	0.4-4.1	1.827(1)	0.313
Unprotected sex	45 (16.7)	12.6-21.5	48 (24.7)	19.1-31.2	4.594(1)	0.032^**^
Unwanted pregnancies	4 (1.5)	0.5-3.5	3 (1.6)	0.4-4.1	0.003(1)	1
Social and interpersonal issues	-	-	-	-	-	-
Damaged property	12 (4.5)	2.5-7.4	9 (4.6)	2.3-8.3	0.008(1)	0.928
Fight with stranger	15 (5.6)	3.3-8.8	15 (7.7)	4.6-12.1	0.884(1)	0.347
Interpersonal conflict	36 (13.3)	9.7-17.8	34 (17.6)	12.7-23.5	1.609(1)	0.205
Safety issues	-	-	-	-	-	-
Drove under the influence	27 (10)	6.8-14.0	45 (23.2)	17.7-29.5	14.995(1)	<0.001^**^
Trouble with police or campus security	1 (0.4)	0.0-1.7	4 (2.1)	0.7-4.9	3.053(1)	0.166
Academic issues	-	-	-	-	-	-
Memory loss	28 (10.4)	7.2-14.4	26 (13.4)	9.2-18.7	1.009(1)	0.315
Missing classes	11 (4.1)	2.2-6.9	10 (5.2)	2.7-9.0	0.305(1)	0.581
Poor academic performance	11 (4.1)	2.2-6.9	14 (7.2)	4.2-11.5	2.187(1)	0.139
Financial strain	-	-	-	-	-	-
Became short on money	44 (16.4)	12.4-21.2	33 (17.1)	12.3-22.9	0.037(1)	0.847

## Discussion

The sample prevalence of current alcohol use within the past 30 days among undergraduates aged 18-25 years was 60.8% (471/775; 95% CI: 57.3-64.2), which was closely aligned with the national estimate of 55.3% (95% CI: 48.2-62.1) reported for persons aged 18-29 years [2]. The sample prevalence of alcohol misuse was 29.1% (225/772; 95% CI: 26.0-32.4), which is notably higher than the national estimate for heavy episodic drinking among the 18-29-year-old group, reported at 19.4% (95% CI: 13.9-26.4) [2]. Heavy episodic drinking is defined as consuming six or more drinks on a single occasion at least once within the past 30 days and closely corresponds to binge drinking, a recognized form of alcohol misuse [2,18].

Age-stratified analysis indicated that students aged 21-25 years exhibited a higher prevalence of alcohol misuse than those aged 18-20 years. Additionally, students aged 21-25 years from UTT reported higher rates of negative alcohol-related consequences experienced within the past year, with unprotected sex and driving under the influence showing significant associations with age group. These findings indicate that older young adults may not be inherently more responsible when it comes to their alcohol consumption.

Public health implications

The age-related pattern observed in this study suggests that alcohol-related prevention efforts should include students aged 21 years and older, not only those below a proposed legal drinking-age threshold. In the UTT dataset, students aged 21-25 years were significantly more likely to report unprotected sexual activity under the influence of alcohol. This behavior may have public health implications, including potential risks related to sexually transmitted infections and unintended pregnancies. These findings should be interpreted as cross-sectional associations and not as evidence of the potential effects of changing the legal drinking age.

The aOR for alcohol misuse among men compared to women was 1.07 (95% CI: 0.74-1.55), indicating no significant gender difference. This is noteworthy given that earlier research conducted about two decades ago among UWI St. Augustine students found that males were more likely to engage in binge drinking [8]. The present cross-sectional study cannot establish a temporal trend; however, the absence of a clear sex-based difference is consistent with previous literature describing growing concern about alcohol misuse among young women [20]. Similarly, in the United States, women over the age of 21 increasingly engage in alcohol use as they progress into their thirties and forties [21]. This shift is of public health concern, as women are more susceptible to alcohol-related health complications, including increased healthcare utilization, mortality, hepatitis, cardiovascular disease, neurological damage, and breast cancer [22]. Public health strategies should extend beyond age-based regulations to encompass the shifting patterns of alcohol misuse among young women.

UTT students aged 21-25 years reported significantly higher rates of driving under the influence within the past 12 months, at 23.2% (45/194; 95% CI: 17.7-29.5), compared to their 18-20-year-old counterparts, at 10.0% (27/270; 95% CI: 6.8-14.0). Road traffic data from 2000-2011 indicate that most collisions in Trinidad and Tobago occurred on weekends, many of which were assumed to involve alcohol or drug use [23]. Individuals aged 15 to 44 years were disproportionately affected, likely due to their greater participation in entertainment-related activities during these periods [23].

The Trinidad and Tobago Police Service (TTPS) statistics in 2024 showed that the majority of road user fatalities were among individuals aged 35-44 years (21%), 25-34 years (20%), 45-54 years (15%), and 15-24 years (11%) [24]. The TTPS did not specify how many drivers were under the influence of alcohol; however, the distribution suggests that individuals aged 18-20 years may be less involved in such incidents, whereas those aged 35-44 years appear to be more significantly affected. This aligns with national data identifying adults aged 30-44 years as having the highest prevalence of current and heavy episodic drinkers [2]. These findings suggest that alcohol-related road safety strategies should not focus solely on individuals below the age of 21, but should also address risky drinking and impaired-driving behaviors among older young adults and the wider adult population.

Heightened activity in the adolescent brain reward and stress-related neural systems, coupled with ongoing prefrontal cortex maturation, which continues into the twenties, may increase vulnerability to alcohol use during this developmental period [25]. This provides compelling support for delaying the initiation of alcohol consumption until post-adolescence, during the twenties. However, curbing early initiation remains a challenge in Trinidad and Tobago, with the legal drinking age set at 18 years; 82.5% (254/308) of UWI students indicated that their first experience with alcohol occurred during primary or secondary school. Therefore, age threshold policies must be supported by comprehensive behavioral and educational interventions, along with complementary policy measures, to effectively address underage initiation [15].

Limitations

This study has several limitations. The cross-sectional design limits causal interpretation and does not allow for the determination of whether age directly influences alcohol misuse or alcohol-related harms. The UWI sample-size calculation was based on e-cigarette prevalence among students aged 13-15 years for the parent study, rather than alcohol-related outcomes, which may affect the representativeness and statistical precision of the present secondary analysis. Alcohol use, alcohol misuse, and alcohol-related harms were self-reported and may be subject to recall bias or social desirability bias. The UTT survey included a convenience sampling component because available classes were selected during campus visits when class schedules were not available for random class selection; this may have introduced selection bias and limited the representativeness of the UTT subsample.

Moderate drinking was not measured identically across the two datasets, as one survey used daily sex-specific thresholds while the other used a weekly threshold. This may have introduced exposure misclassification for moderate drinking and may have affected descriptive estimates for this category. However, moderate drinking was not used as the primary outcome in the regression analysis, and the main alcohol misuse outcome was derived from binge drinking and/or heavy drinking. Some measures were dataset-specific, including AUDIT scores and selected alcohol-related negative effects in the UTT dataset, and age and school at first alcohol use in the UWI dataset. Finally, the findings are limited to undergraduate students from UTT and UWI and may not be generalizable to all young adults in Trinidad and Tobago.

The analyses did not apply survey weights or design-based variance estimation because complete design information, including consistent sampling probabilities, design weights, and cluster identifiers, was not available across the UTT and UWI datasets. This means that confidence intervals and regression standard errors may not fully account for clustering, stratification, unequal probabilities of selection, or differences in institutional source-population size. The findings should therefore be interpreted as unweighted sample estimates and may not be fully generalizable to all undergraduate students or young adults in Trinidad and Tobago.

The UWI and UTT surveys were conducted in adjacent academic years, but institutional differences, survey timing, and unmeasured contextual factors may still have influenced the observed age-related associations. Institution was included as a covariate in the pooled regression model, and institution-specific sensitivity analyses showed similar effect estimates; however, residual temporal or institutional heterogeneity may remain.

Recommendations

Evaluation of existing driving under the influence laws is needed to assess their effectiveness in changing driving-related behaviour. Innovative approaches may be required, such as special identification for designated drivers at major entertainment events, ensuring they are not served alcohol and are subject to breathalyzer testing before departure. The introduction of regulated substitute driver services may be beneficial, as these programs have demonstrated reductions in impaired driving, motor vehicle accidents, and driving under the influence arrests [26].

The WHO SAFER initiative encourages member states to focus on "best buy" strategies to reduce alcohol-related harm by strengthening restrictions on alcohol availability, enforcing countermeasures for drunk driving, facilitating access to screening and treatment, banning or restricting alcohol promotion, sponsorships, and advertising, and raising alcohol prices through taxes and pricing policies [27].

Approximately 48.9% to 54.2% of the adult population are current drinkers [2]; a more equitable and economically beneficial strategy is to increase alcohol prices through taxation. This approach has the potential to decrease expenditure on alcohol, reducing consumption and enhancing government revenue [5]. National campaigns advocating for increased alcohol taxation are estimated to be supported by 87.7% of households [7]. Recent taxation on alcoholic beverages in Trinidad and Tobago [28] has led to early reports of decreased sales of 40-50% in some bars [29].

Educational programmes in tertiary institutions and the workplace should emphasize responsible drinking, harm reduction, strategies to resist peer pressure, and safe sexual practices. These programmes must also be gender-sensitive and highlight gender-specific health risks. Establishing a national alcohol surveillance system would facilitate monitoring of alcohol consumption patterns, misuse, and alcohol-related harms across age groups and genders, thereby informing adaptive policy responses. Confidential screening and referral services for individuals with detected AUD could also be integrated into the surveillance framework.

The current legal age of 18 has not effectively deterred underage alcohol consumption, and alcohol-related issues remain prevalent among students aged 21 years and older. The high sample prevalence of lifetime alcohol use and early initiation indicate that legislative changes must be supported by behavioural and educational interventions [1,6]. Comprehensive alcohol education and prevention programmes at the community level are needed to address early initiation, targeting both parents and children to raise awareness of the risks associated with underage drinking, addiction, developmental harm, and disease.

Legislative support is necessary to discourage the provision and sale of alcohol to minors. In Trinidad and Tobago, 87.4% of households would support policies requiring proof of age at the point of purchase, and 79.5% support holding sellers of alcohol accountable [7]. Mystery shopper interventions have demonstrated effectiveness in promoting responsible alcohol retail practices; these interventions significantly enhance the likelihood that sales personnel will perform age verification when selling alcoholic beverages [30].

Future longitudinal and policy-focused research in Trinidad and Tobago is essential to clarify how legal age limits interact with social and cultural factors shaping alcohol use and to guide evidence-based reforms that address both early initiation and risky drinking behaviours among young adults.

## Conclusions

Alcohol misuse among young adults in Trinidad and Tobago, particularly undergraduate students, should be viewed as a broader public health concern that extends beyond legal drinking-age thresholds. This cross-sectional study identified age-related patterns in alcohol misuse and selected alcohol-related harms but was not designed to estimate the effect of changing the legal drinking age. The findings suggest the need for comprehensive, context-specific public health approaches that address early initiation, risky drinking patterns, and alcohol-related harms among young adults. Future longitudinal and policy-focused research is needed to evaluate how legal age limits interact with the behavioural and cultural factors that shape alcohol consumption in Trinidad and Tobago.

## References

[1] Reid SD, Malow RM, Rosenberg R (2012). Alcohol, drugs, sexual behavior, and HIV in Trinidad and Tobago--the way forward. J Int Assoc Physicians AIDS Care (Chic).

[2] (2025). Country report 2024: Trinidad and Tobago non-communicable diseases risk factor survey (Pan American STEPS). Jaisingh L, Sharma K, James A, Franklin R, Bridgeal J, et al.: Country report.

[3] Ramdeo J (2025). T&T poised to join minority of nations with 21-year drinking age. https://www.guardian.co.tt/news/tt-poised-to-join-minority-of-nations-with-21year-drinking-age-6.2.2352730.bea91beb4f.

[4] (2024). Alcohol. https://www.who.int/news-room/fact-sheets/detail/alcohol.

[5] World Health Organization (2025). World Health Organization: Global status report on alcohol and health and treatment of substance use disorders. World Health Organization: global status report on alcohol and health and treatment of substance use disorders.

[6] Maharaj RG, Motilal MS, Babwah T (2017). National Alcohol Survey of Households in Trinidad and Tobago (NASHTT): alcohol use in households. BMC Public Health.

[7] Maharaj RG, Babwah T, Motilal MS, Nunes P, Brathwaite R, Legall G, Reid SD (2018). The National Alcohol Survey of Households in Trinidad and Tobago (NASHTT): willingness to support changes in policy, laws and regulations. BMC Public Health.

[8] Dhanookdhary AM, Gomez AM, Khan R (2010). Substance use among university students at the St Augustine campus of the University of the West Indies. West Indian Med J.

[9] Castaño-Perez GA, Calderon-Vallejo GA (2014). Problems associated with alcohol consumption by university students. Rev Lat Am Enfermagem.

[10] Mehra D, Agardh A, Stafström M, Östergren PO (2014). Is drinking alcohol associated with sexual coercion among Ugandan university students?: a cross-sectional study. Reprod Health.

[11] McAlaney J, Dempsey RC, Helmer SM (2021). Negative consequences of substance use in European university students: results from Project SNIPE. Eur Addict Res.

[12] Norberg KE, Bierut LJ, Grucza RA (2009). Long-term effects of minimum drinking age laws on past-year alcohol and drug use disorders. Alcohol Clin Exp Res.

[13] Kaestner R, Yarnoff B (2011). Long-term effects of minimum legal drinking age laws on adult alcohol use and driving fatalities. J Law Econ.

[14] Carpenter C, Dobkin C (2025). The minimum legal drinking age and morbidity in the US. https://people.ucsc.edu/~cdobkin/Papers/2016%20The%20Minimum%20Legal%20Drinking%20Age%20and%20Morbidity%20in%20the%20US.pdf.

[15] Roodbeen RT, Dijkstra RI, Schelleman-Offermans K, Friele R, van de Mheen D (2021). Examining the intended and unintended impacts of raising a minimum legal drinking age on primary and secondary societal harm and violence from a contextual policy perspective: a scoping review. Int J Environ Res Public Health.

[16] (2025). Trinidad and Tobago: Global Youth Tobacco Survey 2017. https://extranet.who.int/ncdsmicrodata/index.php/catalog/802/related-materials.

[17] (2025). About standard drinks. https://www.cdc.gov/alcohol/standard-drink-sizes/index.html.

[18] (2026). Understanding alcohol drinking patterns. https://www.niaaa.nih.gov/alcohol-health/overview-alcohol-consumption/moderate-binge-drinking.

[19] (2026). Scoring the AUDIT. https://auditscreen.org/about/scoring-audit/.

[20] Slade T, Chapman C, Swift W, Keyes K, Tonks Z, Teesson M (2016). Birth cohort trends in the global epidemiology of alcohol use and alcohol-related harms in men and women: systematic review and metaregression. BMJ Open.

[21] Keyes KM, Jager J, Mal-Sarkar T, Patrick ME, Rutherford C, Hasin D (2019). Is there a recent epidemic of women's drinking? A critical review of national studies. Alcohol Clin Exp Res.

[22] (2025). Women and alcohol. https://www.niaaa.nih.gov/publications/brochures-and-fact-sheets/women-and-alcohol.

[23] Gopaul CD, Singh-Gopaul A, Sutherland JM, Rostant L, Ebi KL, Chadee DD (2016). The epidemiology of fatal road traffic collisions in Trinidad and Tobago, West Indies (2000-2011). Glob Health Action.

[24] (2025). Arrive Alive Statistics. https://arrivealivett.com/statistics/.

[25] (2025). Alcohol and the adolescent brain. November.

[26] Fell JC, Scolese J, Achoki T, Burks C, Goldberg A, DeJong W (2020). The effectiveness of alternative transportation programs in reducing impaired driving: a literature review and synthesis. J Safety Res.

[27] (2025). SAFER: a world free from alcohol related harm—about the initiative. https://www.who.int/initiatives/SAFER/about.

[28] (2026). Ministry of Finance budget statement 2026 T&T first: building economic fairness through accountable fiscal policies. https://www.finance.gov.tt/wp-content/uploads/2025/10/Budget-Statement-2026-for-web.pdf.

[29] Wilson S (2025). Bar owners say alcohol sales plunge 50% after tax hike. https://www.guardian.co.tt/news/bar-owners-say-alcohol-sales-plunge-50-after-tax-hike-6.2.2442938.a11335f443.

[30] Grube JW, DeJong W, DeJong M, Lipperman-Kreda S, Krevor BS (2018). Effects of a responsible retailing mystery shop intervention on age verification by servers and clerks in alcohol outlets: a cluster randomised cross-over trial. Drug Alcohol Rev.

